# Membranes for Environmentally Friendly Energy Processes

**DOI:** 10.3390/membranes2040706

**Published:** 2012-10-18

**Authors:** Xuezhong He, May-Britt Hägg

**Affiliations:** Department of Chemical Engineering, Norwegian University of Science and Technology, Trondheim NO-7491, Norway; Email: xuezhong.he@chemeng.ntnu.no

**Keywords:** membrane, CO_2_ capture, flues gas, natural gas sweetening, biogas upgrading, hydrogen production, VOC recovery, pressure retarded osmosis

## Abstract

Membrane separation systems require no or very little chemicals compared to standard unit operations. They are also easy to scale up, energy efficient, and already widely used in various gas and liquid separation processes. Different types of membranes such as common polymers, microporous organic polymers, fixed-site-carrier membranes, mixed matrix membranes, carbon membranes as well as inorganic membranes have been investigated for CO_2_ capture/removal and other energy processes in the last two decades. The aim of this work is to review the membrane systems applied in different energy processes, such as post-combustion, pre-combustion, oxyfuel combustion, natural gas sweetening, biogas upgrading, hydrogen production, volatile organic compounds (VOC) recovery and pressure retarded osmosis for power generation. Although different membranes could probably be used in a specific separation process, choosing a suitable membrane material will mainly depend on the membrane permeance and selectivity, process conditions (e.g., operating pressure, temperature) and the impurities in a gas stream (such as SO_2_, NO_x_, H_2_S, *etc.*). Moreover, process design and the challenges relevant to a membrane system are also being discussed to illustrate the membrane process feasibility for a specific application based on process simulation and economic cost estimation.

## 1. Introduction

In the International Energy Outlook 2011 (*IEO2011*) Reference case, world energy consumption is expected to increase by 53% from 2008 to 2035 [1], and the world energy-related carbon dioxide emissions will rise from 30.2 billion metric tons in 2008 to 35.2 billion metric tons in 2020, and 43.2 billion metric tons in 2035, followed by a strong economic growth and continued heavy reliance on fossil fuels. Control of anthropogenic emissions of greenhouse gases (GHG) such as CO_2_ and hydrocarbons (e.g., CH_4_, and volatile organic compounds (VOC)) is one of the most challenging environmental issues related to global climate change. Reduction of CO_2_ emissions from large CO_2_ point sources, especially fossil-fired power plants, based on CO_2_ capture and sequestration (CCS) technology could be a potential approach for the fight against global warming. The key motivation for CCS is that fossil fuels can be continuously used without causing significant CO_2_ emissions, and the captured CO_2_ could be further processed in different ways, such as injected into oil wells and gas fields for sequestration [2], converted to important products such as methanol [3] or producing third-generation biofuels (algae) based on photosynthesis [4]. Development of renewable energy forms such as wind power, solar energy, hydrogen energy, and biogas may become another feasible option for the reduction of CO_2_ emissions. Renewable energy is one of the fastest-growing sources for world energy consumption with a 2.8% increase every year due to the relatively high oil prices, as well as the concern for the environmental impacts of fossil fuel uses and strong government incentives for increasing the use of renewable energy, as reported in IEO2011 [1]. However, in order to satisfy the energy demand for the present and future, the existing alternative energy production technologies must be advanced beyond their current limitations [5], and additional sources of sustainable energy must be explored. Pressure retarded osmosis (PRO) for power generation could be another viable source of renewable energy [6,7]. In addition, the increasing demands of clean and renewable energy have resulted in an increased global willingness to embrace the proposed “hydrogen economy” as a potential long term solution for sustainable development [8].

**Figure 1:**
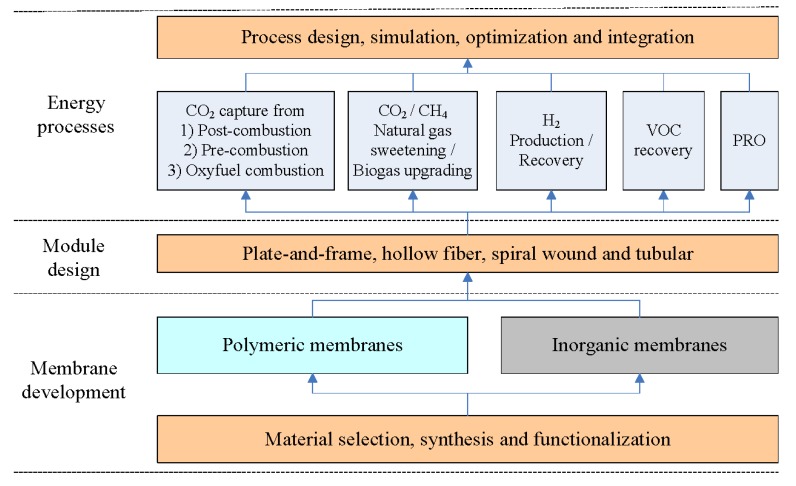
An overview of membrane systems used in different energy processes.

Membranes are becoming a competitive technology compared to the conventional separation unit operations, e.g., cryogenic distillation, chemical and physical absorption. Membrane gas separation has played an important role in various environmental and energy processes, such as CO_2_ capture [9,10,11,12,13,14], VOC recovery [15], natural gas sweetening [16,17], biogas upgrading [18,19], hydrogen production [20,21,22] during the last two decades, and can potentially compete with some traditional separation methods in terms of energy requirements and economic costs. Different types of membrane materials such as common polymers, microporous organic polymers (MOPs), fixed-site-carrier (FSC) membranes, mixed matrix membranes (MMMs), carbon molecular sieve membranes (CMSMs), as well as inorganic (ceramic, metallic, zeolites) membranes, have been reported to be used in various gas separation processes [9,11,12,14,16,23,24,25,26,27,28,29,30,31]. Moreover, proton exchange membranes (PEM) electrolyzers have been used for H_2_ production [32,33,34]. Recently, pressure retarded osmosis (PRO) technology for power generation (based on knowledge about reverse osmosis (RO)) or forward osmosis (FO) membranes show a great potential for sustainable energy production [35,36,37,38]. Figure 1 shows an overview of membrane systems for environmentally friendly energy processes from materials to applications. Choosing a suitable membrane material for a specific application will mainly depend on feed gas composition, process conditions as well the separation requirements. Bernardo *et al.* conducted a review on the status of membrane materials (typically focused on O_2_/N_2_, CO_2_/N_2_ and CO_2_/CH_4_ membranes), relevant industrial applications, and future opportunities [30]. Their contribution addressed the state-of-the-art materials and the major efforts in the development of the membrane gas separation field. In this paper, an extended review of currently used membrane systems for different applications in energy processes has been conducted, and here we focus more on the challenges, process feasibility and economic costs of membrane gas separations.

## 2. CO_2_ Capture from Power Plants

**Figure 2:**
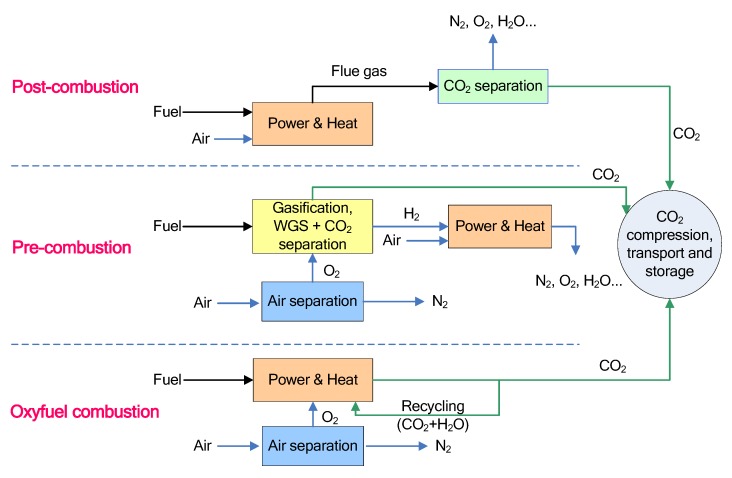
Three options for CO_2_ capture from fossil fuel power plants [39].

The existing fossil fuel power plants for electricity generation without the CO_2_ capture process could be challenging due to the implication of anthropogenic emissions of CO_2_ for global warming. A potential solution to reduce CO_2_ emissions is to develop an efficient CO_2_ capture technology that can be used to retrofit the existing power plants or design a new combustion process with a high efficiency CO_2_ capture unit. Carbon capture and sequestration (CCS) could be an effective way to mitigate the emissions of CO_2_ into atmosphere from fossil fuel power plants, which can be classified as three different scenarios: post-combustion, pre-combustion and oxyfuel combustion as shown in Figure 2 [39]. 

### 2.1. Post-Combustion CO_2_ Capture

Different techniques such as chemical absorption (e.g., MEA, MDEA) and physical absorption (e.g., Selexol, Rectisol), physical adsorption (e.g., molecular sieves, metal organic frameworks) and gas separation membranes can be used for CO_2_ capture from flue gas in post-combustion processes. Membrane separation is energy saving, space saving, easy to scale up, and can be a promising technique for CO_2_ capture as suggested by Yang *et al.* [14]. However, there are some challenges related to the potential applications of membrane systems in post-combustion CO_2_ capture processes as summarized in Table 1 [10,40]. According to these challenges, a low cost, highly CO_2_-permeable, and highly CO_2_-selective membrane is required for a membrane system to compete with a traditional chemical absorption method. Choosing a suitable membrane material is mainly dependent on the process conditions and separation requirements. If high purity of the product is required, a higher selectivity membrane is preferred. If large gas quantities need to be treated, a high permeance membrane will be preferred. 

**Table 1:** Challenges related to standard membrane systems used in post combustion process.

Flue gas characteristic	Challenges related to membrane process	Potential solution	Membrane requirement
Low CO_2_ concentration	Large quantities of gas need to be treated	Scaling up of membrane unit	High CO_2_ selectivity and permeance, low cost
Low pressure	Low driving force	Compression in feed or vacuum in permeate streams	High CO_2_ selectivity and permeance
High temperature	Most polymer membrane cannot be used at >100 °C	Cooling down 40–60 °C	High thermal resistance
Harmful componentsin flue gas	SO_2_, NO_x_	Removal of containments or developing chemically resistant membranes	High chemical and aging resistance
Water	Water can pass through the membranes, corrosion of pipeline during CO_2_ transportation	Drying of flue gas	Low H_2_O/CO_2_ selectivity

A large EU project (NanoGLOWA) was launched in 2006, which mainly focused on the investigation of potential membrane materials for CO_2_ capture from flue gas. The project was based on the cooperation between 27 European companies, universities, institutes and power plants (www.nanoglowa.com), and in 2011, two small pilot-scale membrane modules were installed at EDP’s power plant in Sines (Portugal) and EON’s power plant in Scholven (Germany) to demonstrate the potential of CO_2_ capture using polymeric membranes. The testing in Portugal was performed over ~6 months, and separation properties and durability of the fixed-site-carrier (FSC) membranes were demonstrated in an actual flue gas stream. The performance of this FSC-membrane has since then been greatly improved with a permeance of ~5 m^3 ^(STP)/(m^2^ h bar), and selectivity CO_2_/N_2_ > 1000 [41]. 

Many research activities on the investigations of different membranes for CO_2_ capture have been conducted, some examples are given in [9,10,11,12,13,14]. He *et al.* investigated the application of hollow fiber carbon membranes for CO_2_ capture from flue gas [28,42,43,44]. They reported a capital cost of 100 $/tonne CO_2_ avoided using carbon membrane [42], which is still higher than traditional chemical absorption method of MEA (59 $/tonne CO_2_ avoided reported by Rao and Rubin [45]), but the referred carbon membranes had a clear potential for further optimization. Merkel *et al.* [46] reported that a membrane with a CO_2_/N_2_ selectivity above 50 and a 4000 GPU permeance (1 GPU = 2.736 × 10^−3^ m^3 ^(STP)/(m^2^ h bar)) could offer a capture cost below 15 $/tonne CO_2_, which is lower than the US Department of Energy’s (DOE) target of 20 $/tonne CO_2_ [47]. Their innovative process solution to the CO_2_ capture is also contributing strongly to their conclusion where they also pointed out that improving membrane permeance is more important than increasing selectivity (given selectivity is >30) in order to further reduce the membrane unit’s cost. Hussain *et al.* conducted a feasibility analysis by HYSYS integrated with an in-house membrane program (ChemBrane, developed by Grainger [48]) to investigate the influence of process parameters on energy demands and cost using a novel CO_2_-selective FSC membrane [13]. Their results indicated that a membrane system using high performance FSC membranes was feasible for CO_2_ capture, even at a low CO_2_ concentration (~10%) in a flue gas, compared to amine absorption in terms of energy requirement, and it was also possible to achieve more than 90% CO_2_ recovery and >95% CO_2 _purity in permeate stream. In any case, this environmentally friendly technology with improved membrane performance could promote membrane systems as a promising candidate for CO_2_ capture from flue gas in post-combustion process if all the challenges (shown in Table 1) can be well addressed. 

Liquid membranes have also been investigated for CO_2_ separation [49,50,51,52,53], and most of these works use non-volatile liquids of room temperature ionic liquids (RTILs) as the carriers to transport CO_2_. A review on the development of liquid membranes for gas/vapor separation has been conducted by Krull *et al.* [54]. They pointed out that the use of ionic liquids could improve the liquid membrane stability. However, ionic liquids are still in a minority and not commercially available. Another challenge of liquid membranes is the lack of long-term stability when faced with CO_2_ capture in industrial application. Membrane contactors, which combine gas separation membranes and solvents, offer a unique way to perform gas-liquid absorption and provide high operational flexibility [55]. There has been strong interest focused on the improvement of membrane contactor efficiency for CO_2_ capture [55,56,57,58,59,60,61,62]. Yeon *et al.* [60] reported the use of a polyvinylidene difluoride (PVDF) hollow fiber membrane contactor for absorption and a stripper column as desorber for CO_2_/N_2_ separation, which presented a higher CO_2_ removal efficiency than the conventional absorption column. Feron *et al.* have investigated the potential application of CO_2_ capture from flue gas using a membrane contactor composed of porous polypropylene hollow fiber membranes and a dedicated absorption liquid (CORAL) [59]. Their results indicated that a membrane contactor could be an interesting candidate for CO_2 _capture from flue gas in post-combustion power plants. In conclusion, for post-combustion CO_2_ capture, the indications are that the only membranes which will have a potential of being economically viable will need to be polymers with high flux, and moderate to high selectivity (CO_2_/N_2_) and can tolerate impurities such as SO_2_, NO_x_ and H_2_O. The new membrane contactors containing stable carriers are also promising candidates. Innovative energy-saving process solutions will likewise help to promote membranes for this application.

### 2.2. Pre-Combustion CO_2_ Capture

Pre-combustion CO_2_ capture is often referred to as CO_2_/H_2 _separation at high temperature and pressure. Both CO_2_-selective and H_2_-selective membranes can be used for this application. For a CO_2_-selective membrane, H_2_ will be kept in the retentate stream with high concentration and pressure, and can be directly combusted in the turbine. While for H_2_-selective membranes, CO_2_ will remain in the retentate stream with high pressure and can be conveniently compressed for transportation and storage. While a CO_2_-selective membrane at high temperature is rare for this gas pair, several H_2_-selective membranes have been widely investigated. Membrane is then usually integrated with water-gas shift (WGS) reaction as a membrane reactor for driving the production of H_2_. Scholes *et al.* conducted a comprehensive review on membranes for CO_2_ capture from pre-combustion processes [63]. They reviewed various types of membranes and membrane reactors as well as membrane processes and economics. Here we mainly have focused on the process feasibility of different membranes for pre-combustion CO_2_ capture.

Choosing a suitable membrane material is mainly dependent on process design, operating conditions, and the location of a membrane system in a pre-combustion process since each type of membrane material has its own optimal operating temperature range and limitations. Palladium (Pd) membrane is typically used for H_2 _separation and purification in combination with a reactor in water gas shift (WGS). Pd-membrane has an extremely high selectivity for H_2_ over the other gas molecules. However, Pd membranes may suffer poisoning problems due to interactions with sulfur compounds, CO and unsaturated hydrocarbons which are present in syngas of pre-combustion processes. Some Pd-alloys such as Pd-Au and Pd-Cu membranes have been developed and showed more resistance to sulfur poisoning [64,65]. Pd membranes also undergo phase change below their critical point of 571 K and 2 MPa leading to boundary defects and hydrogen embrittlements [66], which can be partly reduced by adding some other metals, such as Ag, Cu, or Ni. Another challenge of Pd membranes is their short lifetime under harsh conditions, which may hinder their commercial applications [63,66]. 

Ceramic membranes can also be used for high temperature applications, especially in the chemically aggressive environment of a pre-combustion process. However, the main challenge of ceramic membranes is the stability of the selective layer in a hot stream. Polybenzimidazole (PBI) is a thermally stable polymer with a reported glass transition temperature of 420 °C, and PBI-based membranes can be operated at high temperature (200–400 °C). Krishnan *et al.* [67] conducted process simulation of CO_2_ capture from syngas using H_2_-selective PBI membranes in an integrated gasification combined cycle (IGCC) process. They simulated four scenarios including IGCC without CO_2_ capture (scenario 1), CO_2_ capture using Selexol (scenario 2), CO_2_ and H_2_S capture using PBI membranes (scenario 4A), CO_2_ capture using PBI membranes and H_2_S capture using Selexol (scenario 4B). A CO_2_ avoidance cost of 39 $/tonne CO_2_ was found for scenario 4A to attain 90% CO_2_ removal, which is much lower compared to the other scenarios (scenario 2 and scenario 4B are 50 and 54 $/tonne CO_2_, respectively) reported in their simulation results. 

A highly selective CO_2_/H_2_ facilitated transport membrane in an IGCC power plant was also investigated by Grainger *et al.* [68]. Their membranes comprised a thin polyvinylamine (PVAm) selective layer coated on a ultrafiltration (UF) polysulfone support, which showed a superior separation performance with a CO_2_ permeance of 0.1 m^3^ (STP)/(m^2^ h bar), and CO_2_/H_2_ selectivity over 100, based on the mixed gas tests. Their simulation results indicated that the modified process with sour shift process could achieve 85% CO_2_ removal at an acceptable purity for sequestration. The plant cost was calculated to be 2320 €/kW with an electricity production cost of 7.6 € cents/kWh and a CO_2_ avoidance cost of 39 €/tonne CO_2_. The gas separation performance of these FSC membranes has lately been significantly improved as documented in [41] (with a high CO_2_ permeance ~5 m^3 ^(STP)/(m^2^ h bar) and a selectivity >1000 for CO_2_/N_2_), which indicates that the mechanically stronger PVAm/PVA blend FSC membranes could become a promising candidate for pre-combustion CO_2_ capture where pressure is high if the operating temperature can be brought down. 

### 2.3. Oxyfuel Combustion CO_2_ Capture

Oxyfuel (oxygen-enriched) combustion technology provides a promising option based on the combustion using high purity O_2_ produced from an air separation unit (ASU), thus resulting in a flue gas containing mostly CO_2_ and water. Water can then be easily removed via condensation, thereby generating high purity CO_2_ for transportation and storage. One challenge for oxyfuel combustion process is the high combustion temperature with rich O_2_. Habib *et al.* reported to recycle part of flue gas back into the combustion chamber to moderate the combustor temperature [69]. Another challenge is to get the high purity O_2_ source to make oxyfuel combustion process as a competitive CO_2_ capture technology. Conventional O_2_ purification is currently utilizing cryogenic distillation which is an energetically expensive process. An alternative way is to use a membrane system, in which high purity O_2_ can be produced by a two-stage membrane unit. Strong interests have been focused on the development of new membrane materials with high O_2_ permeance and selectivity (O_2_/N_2_) to produce high purity oxygen from air using a single stage membrane unit. Ceramic membranes made from mixed ion-electronic conducting oxides (high temperature ion transport membrane (ITM) [70,71]) have received increasing attention because of their potential to reduce the cost of O_2_ production, which could promote the development of this clean energy process. A commercial ITM membrane module for pure O_2_ production from air has been developed by Air Products [72]. Their ITM oxygen unit can attribute a 48% less capital cost and a 68% energy saving compared to cryogenic ASU. Moreover, pressurized oxyfuel combustion systems could be another potential solution to achieve a high purity CO_2_ in flue gas and reduce the energy penalties, which provides a better performance over conventional atmospheric oxyfuel combustion power cycles [73,74]. CANMET Energy Technology Centre and ThermoEnergy Corp. have conducted the techno-economic evaluations on the pressurized oxyfuel combustion systems [75,76,77]. Their results showed an improved net efficiency and the reduction of capital and electricity costs using high pressure oxyfuel combustion technology. The oxyfuel combustion is a very smart way of capturing CO_2_. The main challenge, however, seems to be the development of a good module and sealing design based on the ceramic oxides which can tolerate the high operating temperatures without leakage and cracks.

## 3. Natural Gas Sweetening

CO_2_ removal from natural gas (natural gas sweetening) is mandatory to meet the specifications of a natural gas grid since CO_2_ reduces the heating values of natural gas, is corrosive, and easily forms hydrates to clog equipment or damage pump [30]. Choosing a suitable technology for CO_2_ removal from natural gas is mainly dependent on process conditions and crude natural gas composition. Traditional chemical (amine) absorption is well known and has been commercially used for CO_2_ removal in various processes, and is still considered as a state-of-the-art technology. However, a membrane system possesses many advantages such as small footprint, low capital and operating costs, is environmentally friendly and exhibits process flexibility [29]. It shows a great potential for natural gas sweetening even though it has only 5% of the market today. Two key parameters—membrane unit cost and CH_4_ loss—are mainly dependent on the membrane performance and process design, which are usually employed to evaluate the efficiency of a membrane process. Cellulose acetate is still widely used in UOP’s membrane system [16] and, recently, Cynara-NATCO installed a cellulose triacetate membrane system using 16-inch hollow fiber modules in Thailand [78]. Although common polymer membranes for natural gas sweetening are still using cellulose acetate/triacetate and polyimide, the novel, high performance composite FSC membranes showed great potential for CO_2_/CH_4_ separation [79]. Membrane systems are preferred for high CO_2_ concentration gas streams (enhanced oil recovery, *ca.* 50% CO_2_, and high pressure) and amine units are preferred for relatively low-concentration gas streams. Moreover, membrane systems are also favorable for processing small gas flows (typically for offshore platforms, <6000 Nm^3^/h) because of their simple flow schemes, while amine units are more complex and require careful, well-monitored operating procedures, as documented by Baker *et al.* [16]. 

High pressure operation is the main challenge for natural gas processing with membrane systems. Plasticization is indeed always a limited factor for high pressure CO_2_ rich gas to be separated with membranes [80,81], while for FSC membranes, carrier saturation at a high CO_2_ concentration and low water content in high pressure feed gas stream cause a significant decrease of CO_2_ permeance as well as selectivity of CO_2/_CH_4 _due to reduced contribution by the facilitated transport mechanism. The possible strategies to overcome membrane plasticization are crosslinking of membrane material [82] and fabrication of mechanical strength enhanced membranes, such as the mixed matrix membrane by adding inorganic fillers to the polymer matrix. Adams *et al.* prepared a 50% (vol.) Zeolite 4A/poly (vinyl acetate) (PVAc) MMM for CO_2_ separation from natural gas [83]. They found that the prepared MMMs can approach the Robeson CO_2/_CH_4_ upper bound, and at low CO_2_ partial pressures, CO_2_ permeability roughly doubled with a nearly 50% increase in selectivity versus pure PVAc under the same conditions. While at high CO_2_ partial pressure, CO_2_ permeability remained effectively unchanged with a 63% increase in selectivity comparing to pure PVAc. Their membranes showed promise for application in high pressure natural gas sweetening. He *et al.* reported that carbon nanotubes (CNTs) reinforced with the PVAm/PVA blend FSC membrane presented a good CO_2/_CH_4_ separation performance with a CO_2_ permeance of 0.11 m^3^ (STP)/(m^2^ h bar) and a CO_2_/CH_4_ selectivity of 22 at 30 bar [84]. It shows a more secure mechanical strength to maintain a good separation performance even at high pressure. 

Process design for CO_2_ removal by membrane system from natural gas depends on the membrane permeance and selectivity, CO_2_ concentration in feed stream, specific separation requirement, as well as the location of the plant. Peters *et al.* conducted process design, simulation, and optimization for CO_2_ removal from natural gas using HYSYS integrated with an in-house membrane programme [17]. They reported that a two-stage membrane system with a CO_2_ permeance 0.3 m^3^ (STP)/(m^2^ h bar) and a CO_2_/CH_4_ selectivity 40 is comparable to that of an amine process. Although the CH_4_ purity (98%) of the sweet gas is lower compared to amine method (99.5%), it can achieve n gas sales standard (<2% CO_2_ in natural gas). However, CO_2_ purity (90%) in the permeate stream needs to be further improved for pipeline transportation and storage since 10% non-CO_2_ gas is needlessly compressed and will cost extra energy—this can be achieved by process design and optimization. A combination of hybrid process comprising a membrane system for bulk removal of CO_2_ from crude natural gas feed with an amine unit for final purification to reach the pipeline specification (<2% CO_2_) was designed by Bhide *et al.* [85]. Baker *et al.* also pointed out that a combination of a membrane system with an amine unit could offer a low-cost alternative to all-amine or all-membrane plants [16]. The future direction of natural gas sweetening using membrane systems will be the development of high performance membranes with an active layer on the order of 0.1 μm in order to compete with other separation methods. In addition, membranes should also have to be: resistant to warm and high pressure operating conditions and mechanically strong. Membrane plasticization and long-term compaction at high pressure should be further investigated. Moreover, how to predict the long-term performance in commercial application based on the short-term lab-scale tests could be a continuing challenge of a membrane system for high pressure natural gas sweetening. A few studies have been conducted on this issue where the researchers are studying the long-term performance of a polymeric PVAm/PVA membrane when exposed to H_2_S, MEG, TEG and higher hydrocarbons (HHC) which are usually present in natural gas. Their membranes seemed to tolerate the impurities relatively well, but were vulnerable to HHC [86,87].

## 4. Biogas Upgrading

Biogas is considered to be one of the most efficient means of utilizing renewable energy and reducing greenhouse gas emissions. The composition of biogas varies depending on the origin of the anaerobic digestion process, and the main components are methane (CH_4_) and carbon dioxide (CO_2_) as shown in Table 2 [88].

**Table 2:** Typical biogas composition from different sources [88].

Process	Composition (vol %) *					H_2_S/SO_2_ (ppm)
	CO_2_	CH_4_	N_2_	O_2_	H_2_O	
Farm biogas plant	37–38	55–58	<2	<1	4–7	32–169
Sewage digester	38.6	57.8	3.7	0	4–7	62.9
Landfill	37–41	47–57	<1	<1	4–7	36–115

* Siloxane is not included.

Biogas can be used as a renewable energy source for heating, vehicle fuel, combined heat and power (CHP) generation, fuel cell and substitute natural gas. However, depending on the different end uses, specific biogas treatment should be executed. For applications such as vehicle fuel and natural gas grid injection, the acid gases of CO_2_ and H_2_S should be removed from raw biogas, *i.e.*, biogas upgrading. High content of CO_2_ in biogas will cause the risks of corrosion of pipeline and decrease the Wobbe index which is directly proportional to the methane concentration. Therefore, CO_2_ removal from natural gas is mandatory in all natural gas processing plants. However, biogas upgrading processes will add extra costs to biogas production, so it is important to find an optimized upgrading technology in terms of lower energy consumption and higher efficiency. Moreover, methane losses during upgrading should be minimized since methane has a greenhouse effect around 24 times higher than that of CO_2_.

Different techniques such as pressure swing adsorption (PSA), physical absorption (e.g., water scrubbing) [89], chemical absorption (e.g., amines) [90,91] and membrane separation [18,19] can be used for biogas upgrading. The choice of a suitable technology is mainly dependent on the specific conditions at a plant, such as availability of low price for heating, electricity and water, as well as the amount of gas to be handled. Today, most biogas upgrading plants in Sweden are using PSA. The upgraded gas has a typical methane concentration around 96% while methane loss is quite high (3%–10%). Plants using water scrubbing will produce a lot of waste water, and electricity consumption is also quite high. Membrane systems could be favorable for biogas upgrading due to a series of advantages, including safety and simplicity of operation, and easy maintenance and operation without hazardous chemicals [18]. Compression of upgraded biogas may vary depending on whether it goes to natural gas grid or will be used for vehicle fuel. Biomethane for vehicle fuel must be compressed up to around 200 bar, while the pressure can be lower if injected into a natural gas pipeline network (<80 bar). The main challenge for a membrane system is pre-treatment of biogas to remove H_2_S and water vapor to protect the membranes, especially for sewage treatment plants and landfill sites where the produced biogases contain high number of malicious gas components such as siloxanes (siloxanes will be a serious problem for some polymeric membranes, e.g., PVDF). Deng *et al.* reported to use composite FSC membranes for biogas upgrading [19]. Their results indicated that membrane process with a CH_4_ recovery of 99% at a low operation cost could be designed to achieve natural gas grid specification, which made this environmentally friendly technique more competitive compared to the other conventional technologies currently used. Makaruk *et al.* pointed out that a membrane system provides enough flexibility for heat integration within biogas plants [18]. The expected energy requirement for a single produced cubic meter of natural gas substitute is around 0.3 kWh, which is close to the values that were reported in an industrial scale technology demonstration for membrane biogas upgrading plant at Bruck/Leitha in Austria [92]. Moreover, a new carbon membrane company MemfoACT (www.memfoact.no) was launched in 2008 in Norway, which mainly focuses on biogas upgrading using carbon membranes. Their contributions could be promising to bring this technique into commercial application in the near future. 

## 5. Hydrogen Production/Recovery

**Figure 3:**
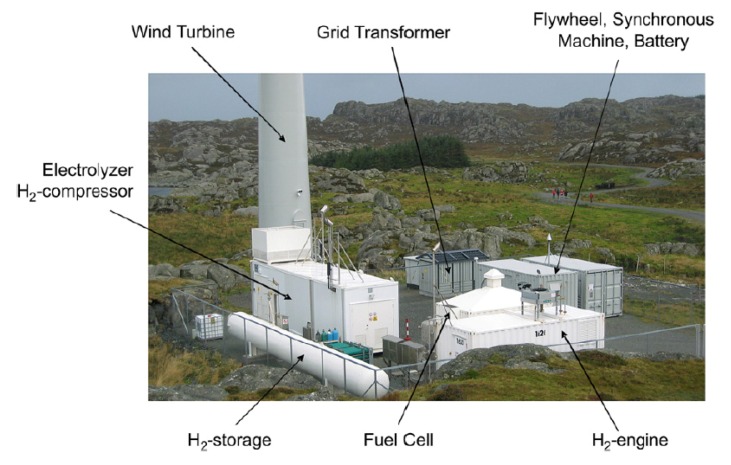
Utsira wind/hydrogen demonstration plant based on proton-exchange membrane (PEM) electrolyzer [21].

Hydrogen energy composes the promise of zero emissions as well as energy independence and safety in the transportation sector, which can be produced and recovered from different processes using membrane systems, as summarized in Table 3. Hydrogen can be generated from a readily available source: water electrolysis based on a proton-exchange membrane (PEM). PROTON developed a PEM electrolyzer FuelGen^®^ to produce high purity hydrogen fuels [20]. Norsk Hydro built a wind/hydrogen energy demonstration system using a PEM electrolyzer and fuel cells at the island of Utsira in Norway 2004, as shown in Figure 3 [21]—this is, however, no longer operational. This system provided two to three days of full energy autonomy for 10 households on the island, and was the first of its kind in the world as reported by Ulleberg *et al.* [93]. 

**Table 3:** Main applications of membrane system for H_2_ production and recovery.

Separation	Process	Membrane	Status
H_2_ production by water electrolysis	H_2_ PEM electrolyzer	PEM, FuelGen^®^	Commercial production
Wind/H_2_ power system	PEM electrolyzer and fuel cells	PEM	Pilot-scale demonstration
H_2_/CO	Methanol steam reforming membrane reactors	Pd and CMS membrane	Lab-scale
H_2_/CO	Adjustment of H_2_/CO ratio in syngas	Silicon rubber, polyimide	Plant installed
H_2_/N_2_	Ammonia purge gas	Prism^®^	Plant installed
H_2_/Hydrocarbon	H_2_ recovery in refineries	Silicon rubber, polyimide	Plant installed
H_2_/CH_4_	Natural gas network transportation	Carbon molecular sieve membranes	Lab-scale

Hydrogen can also be produced from some industrial processes using membrane systems. The first commercial application of a membrane system for gas separation is hydrogen recovery from the ammonia purge gas using Prism^®^ system produced by Air Products [94]. Hydrogen has a very high permeance compared to other gases such as nitrogen and argon, and the high purge pressure (136 bar) in the ammonia process provides enough driving force for gas permeation. This system can achieve a 95% hydrogen recovery, and the recovered hydrogen can reach a high purity of 98% either for the recycle to synthesis loop or in other processes. Industrial hydrogen recovery in refinery plants is mainly carried out by pressure swing adsorption (PSA) and cryogenic separation, while recent membrane systems attract great interest in this area due to their low capital cost and low energy demands. Brunette *et al.* conducted a review on the comparison between PSA, cryogenics and membrane systems for H_2_ recovery from refineries based on their process flexibility, reliability, ease of response to the variations, and expansion capability [95]. They concluded that choosing a suitable technique will mainly depend on feed composition, feed pressure, product flow rate as well as the requirements of product purity. The membrane system showed a lower energy intensity and smaller footprint compared to the other two processes. Recently, H_2_ production using two types of membranes (Palladium (Pd) and carbon molecular sieve (CMS) membranes) in a methanol steam reforming membrane reactor (MR) was reported by Sá *et al.* [22]. Their results indicated that CMS membranes presented higher permeability, higher hydrogen recovery, and lower selectivity, while Pd membranes were more expensive but exhibited much higher selectivity towards hydrogen. A combined CMS + Pd membrane reactor revealed some advantages compared to either CMS-MR or Pd-MR.

If a hydrogen energy-based society is realized, a hydrogen distribution system must be built for hydrogen transportation which will take a long time. A feasible solution is to use the existing natural gas pipeline networks to transport a H_2_ and natural gas mixture, which was proposed by NaturalHy project (6th EU framework) [96]. In that project, Grainger *et al.* studied the separation performance of H_2_/CH_4 _with carbon molecular sieve membranes based on experiments and process simulation [97]. Their techno-economic evaluation results indicated that carbon molecular sieve membranes can offer a great potential for hydrogen separation from hydrocarbon, and high purity hydrogen can be recovered from leaner streams in natural gas networks with a low energy consumption. 

## 6. Volatile Organic Compounds Recovery

**Figure 4:**
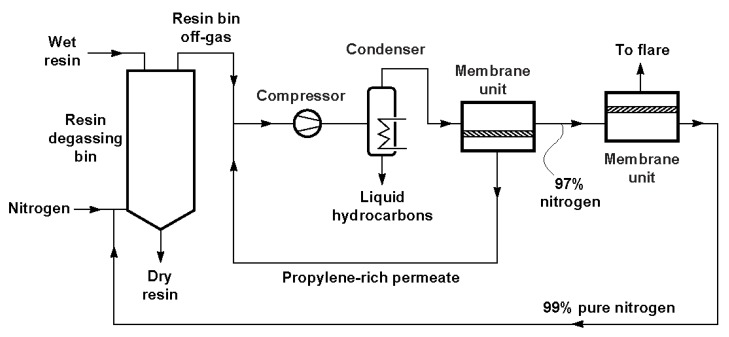
A schematic process flow diagram of a membrane propylene recovery system [15].

Volatile organic compounds (VOC) might be recovered, instead of being released to atmosphere since some of these compounds are involved in atmospheric pollutions and are strong greenhouse gases. Different techniques such as condensation, absorption, adsorption and vapor permeation, *etc.*, can be used for VOC recovery [98]. Among them, vapor permeation membranes attract great interest for VOC recovery from gas streams in various industrial processes, such as polyolefin plant resin degassing and gasoline vapor recovery in large retail gasoline stations [15,99,100]. The main application of vapor separation membranes is the recovery of hydrocarbon monomers from ethylene and polyethylene and polypropylene plants. Following the development of vapor/gas separation membranes, more and more polyolefin plants have installed hydrocarbon recovery units. A schematic process flow diagram of a typical membrane system integrated into a polyolefin plant is shown in Figure 4 [15]. The vapor-enriched permeate stream is recycled to the compressor’s inlet, while high purity N_2_ is sent to the second stage membrane unit for further purification. Membrane Technology and Research, Inc. (MTR) developed a VaporSep^®^ system for propylene recovery from polypropylene (PP) production plants, which has been installed in many petrochemical plants around the world during the last 10 years. 

Gasoline vapor recovery is becoming another important business for membrane gas separation systems. Many gasoline retail stations have installed membrane systems to recover hydrocarbon vapor when it was transferred from trucks to tanks. Some representative companies such as GKSS [101] and MTR [102] have developed gasoline vapor recovery systems for reduction of hydrocarbon emissions. The OPW Vaporsaver^TM^ system [103], fitted with MTR’s membranes, is used for recovery of gasoline vapors and can reduce hydrocarbon emissions by 95%–99%. 

## 7. Pressure Retarded Osmosis

**Figure 5:**
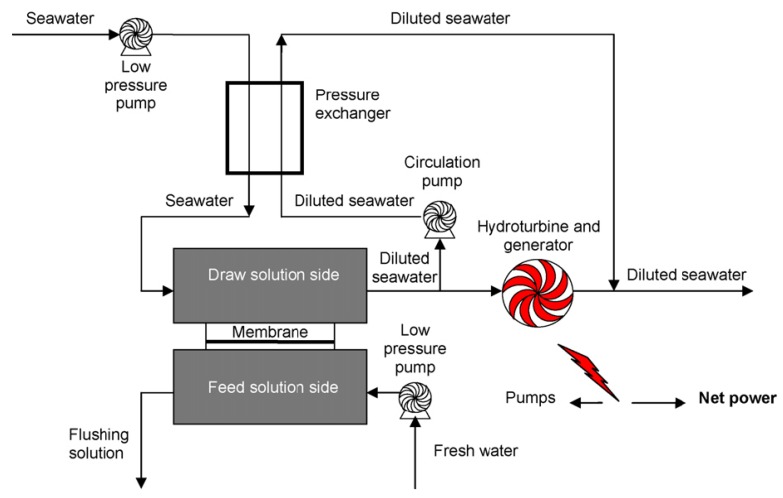
A schematic diagram for pressure retarded osmosis (PRO) power generation system [35].

Pressure retarded osmosis (PRO) has the potential to produce renewable energy from natural and anthropogenic salinity gradients [7]. In a PRO system, water from a low salinity solution permeates through a membrane into a pressurized, high salinity solution; power is generated by depressurizing the permeate through a hydroturbine, as shown in Figure 5 [35]. The concept of energy production from the mixing of fresh water and salt water was first proposed by Pattle [104]. The continuous availability of both natural water resources (sea water) and anthropogenic waste streams showed a great potential of PRO technology for renewable energy production. However, development of PRO technology has been hindered due to the lack of a suitable membrane. Traditional reverse osmosis (RO) membranes cause a severe internal concentration polarization (ICP) phenomenon, which could decrease the water flux significantly. Thus, RO membranes can only achieve a low power density (power produced per membrane area) in a PRO operation unit. Another type of membrane: forward osmosis (FO), suffers less ICP influence, but the relatively low water flux also restricts a PRO system from attaining a high power density [35,36]. Statkraft built the world’s first prototype osmosis power plant in Norway to demonstrate PRO technology (power density <0.5 W/m^2^) [105], and today’s membranes can produce close to 3 W/m^2^. Although power density is still lower than the requirement for commercial viability (power density of 4–6 W/m^2 ^[106]), development of higher performance FO membranes could promote PRO technology to be commercialized for renewable energy production in the near future. 

## 8. Future Directions

Membrane technology shows strong potentials in various energy processes including CO_2_ capture from flue gas in power plants, natural gas sweetening, biogas upgrading, H_2_ recovery, VOC recovery as well as power PRO generation. However, for any kind of suitable application, a high performance membrane material is required, while taking into consideration some key parameters such as transport properties, durability and mechanical strength. The following aspects can be further investigated to achieve a high efficiency membrane process:

- Membrane transport properties (pemeance and selectivity)
- Mechanical strength, chemical and thermal stability under a specific operating condition
- Membrane durability over the long term by being exposed to real process conditions
- Membrane module design
- Process design, simulation, optimization and integration

Three specific parameters—environmental, economic and social indicators—are usually employed to compare membrane systems with the other traditional unit operations towards sustainability, and evaluate which technique could be more suitable for a specific application. However, for any specific application, process conditions need to be carefully considered before making a decision. Nevertheless, the prediction is that membrane systems, which require no or very little chemicals compared to standard unit operations, in addition to being easy to scale up and having low energy consumption, will be an environmentally friendly technology for application in energy processes in the future.
